# News and Coming Events

**Published:** 1909-01-09

**Authors:** 


### NEWS AND COMING EVENTS.

THE following gentlemen have been appointed to the Registrarships at the Middlesex Hospital :—Medical, Mr. Otto May, M.A., M.B., B.C. (Cantab.), M.R.C.P. (Lond.); surgical, Mr. Cecil W. Rowntree, M.B., B.S., F.R.C.S.; and obstetric, Mr. Frank E. Taylor, M.A., M.D., F.R.C.S.

THE annual dinner of the West London Medico-Chirurgical Society will take place at the Wharncliffe Rooms, Great Central Hotel, on Friday, February 12, at 7.30 for 8 P.M. punctually. Communications should be made to Mr. Aslett Baldwin, the honorary secretary to the dinner committee.

THE next meeting of the Society for the Study of Inebriety will be held in the rooms of the Medical Society of London, 11 Chandos Street, Cavendish Square, W., on Tuesday, January 12, 1909, at 4 P.M. (afternoon meeting), when Professor Arthur R. Cushny, A.M., M.D., C.M., F.R.S., will open a discussion on "The Action of Alcohol." Each member and associate is at liberty to introduce a visitor. Tea and coffee at 3.45; council meeting at 3.30.

THE recent death of Mr. B. W. Levy removes one who was a generous and discriminating friend to several hospitals and other medical charities. The large legacy which he received at the death of Mr. David Lewis he devoted entirely to charitable undertakings, which included the rebuilding of the David Lewis Northern Hospital at Liverpool and the founding of the large Colony for Epileptics at Sandlebridge in Cheshire. He also gave £15,000 anonymously to the London Hospital for the building of five new operating theatres.

THE Queen recently sent to the military medical authorities in Ireland a large number of special Christmas gifts for distribution in the military hospitals in the Irish Command which are staffed by ladies of the Queen Alexandra's Imperial Military Nursing Service. Her Majesty's gifts include such articles as pictures, carpets, rugs, and flower vases for decorative purposes in the hospital wards. Surg.-Gen. G. D. Bourke, C.B., with the approval of Sir Neville Lyttelton and acting in consultation with the matrons of the Queen Alexandra's Imperial Military Nursing Staff serving in Ireland, has arranged for the distribution of the Queen's gifts to the wards of all the principal military hospitals in Dublin, the Curragh, and Cork, where they will add materially to the comfort of the patients.

A REUTER despatch from Georgetown, British Guiana, dated November 28, says that arrangements have been completed between the Secretary of State for the Colonies and the Government of British Guiana to enable Professor Deycke, late Director of the Military School of Medicine at Constantinople, to carry out a practical test of his Nastine treatment for the cure of leprosy at the colony leper asylum at Mahaica. The experiment is considered to be of the utmost importance, for, should it prove the success Professor Deycke confidently hopes, it is anticipated that the Imperial Government will take steps actively to encourage the adoption of the remedy in other British colonies. The new treatment was first officially brought to the notice of the Secretary of State by Sir Patrick Manson in the early part of the year, in a letter suggesting that Professor Deycke should be given an opportunity to make a practical and exhaustive trial of his remedy at one of the colonial leper asylums, and the institution at Mahaica in British Guiana was mentioned as offering special facilities for the purpose.

AMONG the survivors of the recent terrible earthquake at Messina is Professor San Felice, the famous cancer specialist, but, according to the correspondent of the *Morning Post*, his scientific collections are completely lost.

ON Tuesday, January 12, at 2.30 P.M., Princess Louise Duchess of Argyll, will attend a matinée of children's plays in aid of the Prince of Wales's General Hospital, Tottenham, and the Kensington District Nursing Association, at the Theatre Royal, Haymarket.

DR. RICHARD CHARLTON HARRISON, who died recently at Ealing, became qualified as a member of the Royal College of Surgeons in 1870, and as a licentiate of the Royal College of Physicians and of the Society of Apothecaries in 1872. He received his medical education at St. George's Hospital, and subsequently became assistant curator to the hospital museum. He was Hon. Local Secretary and Treasurer to the Royal Medical Benevolent College, Medical Officer in the Civil Service, and Surgeon to the Ealing Cottage Hospital.

THE annual meeting of the Association of Public School Science Masters is to be held on January 12th at Merchant Taylors' School, Charterhouse Square. An address on the relation of general to technical science teaching will be given at 11.15 A.M. by the President (Sir Clifford Allbutt, K.C.B., M.D.), and Mr. M. D. Hill (Eton) will read a paper on "Anthropometry in Schools." Subsequently a discussion will take place on science curricula in public schools, and Mr. C. I. Gardiner (Cheltenham) will speak on "The refusal of the General Medical Council to recognise public schools as institutions where medical education may be commenced."

FOLLOWING the news of the death of Dr. T. Lambert Hinton, recently reported at the age of 100 years, a letter has been received by the secretary of the Royal College of Surgeons of England from Surgeon-Major Henry Benjamin Hinton, the oldest living member of the College. Surgeon-Major Hinton, who is residing at Glenelg, South Australia, was born at Portsmouth on March 7, 1813. He became a member of the College of Surgeons in May 1835, and in 1838 joined the Bengal Medical Staff, and served as a surgeon in the 32nd "National Infantry" for thirty years. A congratulatory letter has been sent to Surgeon-Major Hinton by the Royal College.

THE report for 1908 of the University of Edinburgh states that during the past year the total number of matriculated students (including 595 women) was 3,328. Of these 1,490 (including 59 women) were enrolled in the Faculty of Medicine. Of the students of medicine 667, or nearly 45 per cent., belonged to Scotland; 281, or 19 per cent., were from England and Wales; 132 from Ireland; 76 from India; 306, or about 20½ per cent., from British Colonies; and 28 from foreign countries. These figures show that the proportion of non-Scottish students of medicine is well maintained; while the number of Colonial students exceeds by 34 the highest number reached at any time during the last 20 years. The following medical degrees were conferred during 1908: Bachelor of Medicine and Master of Surgery, 4; Bachelor of Medicine and Bachelor of Surgery, 184 (including 19 women); Doctor of Medicine, 76 (including 3 women). The total annual value of the University Fellowships, scholarships, bursaries, and prizes now amounts to about £18,660, of which the Faculty of Medicine receives £3,730.

As was foreshadowed in the Treasurer's report at the annual meeting of the League of Mercy, a cheque for the large amount of £19,000 has been handed over to the King Edward's Hospital Fund for London by the League.

The Treasurers of the Middlesex Hospital have received the sum of £100 from Miss Fanny M. Marriage, being the result of her sale of jam and pickles for the benefit of the Cancer Ward Fund.

The late Mr. Harry Barnato's will confirms the report previously published that £250,000 had been bequeathed to "build, equip, and endow a home, hospital, or other charitable institution," in memory of his brother, Barney Barnato, and his nephew, Woolf Joel.

For the position of county analyst of Surrey, which was held for many years by the late Sir Thomas Stevenson, M.D., over 100 applications have been received, and the General Purposes Committee have selected six gentlemen who will attend before the Surrey County Council next week, when the appointment will be made.

A new in-patient maternity department has been opened at the Middlesex Hospital, consisting of two lying-in wards and a labour ward. The department embodies the latest scientific improvements in ward construction, several new features having been introduced. The students and nurses of the hospital will now be able to see midwifery practised under improved conditions, and on the completion of their training carry their ideas into practical effect in dealing with the cases which come under their care.

The Medical Officer of Health for Islington (Dr. A. E. Harris) reports that in the year just closed the birth rate was 24.35 per 1,000 inhabitants. The death rate from all causes was 13.15 per 1,000, the death rate from epidemic diseases 1.01 per 1,000, and the infantile mortality rate 103 per 1,000 births. These rates are the lowest hitherto recorded in Islington, and when the size of the population, nearly 350,000 persons, as well as its density, is taken into consideration, the mortality figures must be judged exceedingly good.

News has been received in Jersey of the death in India of Dr. Douglas Argyll Robertson, the well-known ophthalmic surgeon. Dr. Robertson left Jersey in November on a trip to India. He intended to return in April to Jersey, where he had been living since his retirement from practice in Edinburgh. Dr. Robertson took the degree of M.D. at St. Andrews University in 1857, and the Fellowship of the Royal College of Surgeons of Edinburgh in 1862 while the LL.D. of the University of Edinburgh was conferred upon him in 1896. His name has long been a household word in the medical profession, more especially in connection with the pupil phenomena of tabes, while for many years, during his tenure of the University Lectureship on Diseases of the Eye, he was a familiar figure to Edinburgh medical students. Dr. Robertson was successively Surgeon Oculist in ordinary to Queen Victoria in Scotland, and Honorary Surgeon Oculist to the King in Scotland. He was president of the International Ophthalmic Congress in 1894 and hon. president in 1888 and 1899, while for many years he was ophthalmic surgeon to the Edinburgh Royal Infirmary. He had also been president of the ophthalmic section of the British Medical Association of the Ophthalmic Society of the United Kingdom and of the Edinburgh Medico-Chirurgical Society while he was a Fellow of the Royal Society of Edinburgh and an honorary member of many foreign learned societies.

The City of London Lying-in Hospital has received £2,000 from the executors of the late Mrs. A. A. Chippendall Higgin.

The Essex Parliamentary Committee has approved of an increase of salary from £700 to £800 per annum of Dr. Ambrose, Coroner for the Metropolitan District, as from January 1 last.

Mrs. Blackston, aged 38, wife of a gardener and florist, of Wandsworth, gave birth on Sunday last to four children. The first birth took place at ten o'clock in the morning and the last at seven o'clock in the evening. The children were then all living. The first died soon after the birth of the last and the last lived till two o'clock next morning. The other two had died meanwhile.

The New Year entertainment to patients of the Charing Cross Hospital was given on the evening of January 5 by the medical and nursing staff. The great hall was decorated for the occasion, and here there was a large company of visitors. At St. Bartholomew's Hospital the patients were entertained as usual in the Great Hall on the afternoon of January 5, and the staff and friends of the hospital on the evenings of January 6 and 7. Members of the Amateur Dramatic Club of the hospital gave a representation of Pinero's farce "Dandy Dick," and the Hospital Musical Society also provided excellent entertainment.

A Conference on Tuberculosis is to be held at Caxton Hall, Westminster, on February 16, 17, 18, and 19. One of the principal means to be adopted for the checking of phthisis will be the education of the public generally in the causes, prevention and treatment of consumption. The conference is under the patronage of the Duchess of Norfolk, the Duchess of Sutherland, Lord Carrington, the Bishop of London, the Lord Chief Justice, Lord Cross, Lord Barnard, and many others. A number of experts on tuberculosis are supporting the movement, and will take part in the discussions, the subjects of which will include "Sanatoria for the Working Classes," "Open-air Schools," "Meat as a Source of Infection," "Breathing," "Infection from Animals to Man," "Milk and Tuberculosis," "The Ventilation of Public Places," and "Châlets for the Consumptive Poor." An exhibition of models of sanatoria, nursing appliances, healthy and unhealthy rooms compared, foodstuffs, etc., will add to the value of the conference. All particulars may be obtained from the secretary, 12 Cavendish Mansions, Portland Place, W.

### THE MEDICAL DIRECTORY FOR 1909.

Messrs. J. and A. Churchill's "Medical Directory" for 1909 is as accurate and as compendious as ever. The total number of names for 1909 is 39,992, as against 39,703 in the previous edition, being an increase of 289. The number of names in the London list for 1909 is 6,420, as against 6,550 in 1908. The provincial list for England contains 17,354 names, as against 17,211, while Wales has 1,394 names for 1909 as against 1,256, and Scotland has 3,845 as against 3,829. The Irish list for 1909 contains 2,656 names as against 2,660 in 1908, while practitioners resident abroad number 5,009 names as against 4,927, and the Navy, Army, and Indian Medical Services show 3,303 entries as against 3,259 in 1908. The twenty-two pages devoted to an Abstract of the principal laws affecting the medical profession remain a model of accuracy, lucidity, and condensation. "The Medical Directory" for the present year is in every way a worthy successor to the sixty-four previous annual editions. The price is, as usual, 14s. net to non-subscribers.

